# Strategies and First Advances in the Development of Prevascularized Bone Implants

**DOI:** 10.1007/s40610-016-0046-2

**Published:** 2016-08-15

**Authors:** Christoph Rücker, Holger Kirch, Oliver Pullig, Heike Walles

**Affiliations:** 1 Translational Center Würzburg ‘Regenerative therapies in oncology and musculoskeletal diseases’, Würzburg Branch of the Fraunhofer-Institute Interfacial Engineering and Biotechnology, IGB, Würzburg, Germany; 2 Department Tissue Engineering and Regenerative Medicine (TERM), University Hospital Würzburg, Würzburg, Germany

**Keywords:** Animal models, Bone regeneration, Prevascularized implant, Decellularization

## Abstract

Despite the great regenerative potential of human bone, large bone defects are a serious condition. Commonly, large defects are caused by trauma, bone disease, malignant tumor removal, and infection or medication-related osteonecrosis. Large defects necessitate clinical treatment in the form of autologous bone transplantation or implantation of biomaterials as well as the application of other available methods that enhance bone defect repair. The development and application of prevascularized bone implants are closely related to the development animal models and require dedicated methods in order to reliably predict possible clinical outcomes and the efficacy of implants. Cell sheet engineering, 3D-printing, arteriovenous loops, and naturally derived decellularized scaffolds and their respective testings in animal models are presented as alternative to the autologous bone graft in this article.

## Introduction

Given optimal conditions, bone fractures in humans heal within 20 weeks. The duration of the regeneration process depends on many different aspects: the severity of the wound, the presence of an open fracture, the amount of bone fragments, associated damage to the blood vessel system or the surrounding soft tissue and the treatment of the bone defect [1].

Depending on the extent and the prompt treatment of a distinction is possible between primary and secondary bone healing [2]. For the primary bone healing, a prerequisite is that the periosteum is not damaged and the fracture ends are in direct contact to each other [3]. Osteoprogenitor cells from the periosteum as well as the endosteum infiltrate the thin zone of connective tissue between both fracture ends and form osteons, thus regenerating the bone [4]. Secondary bone healing occurs if the periosteum, the compact bone, and the bone marrow are damaged and the fracture is not well stabilized. The healing process is initiated by extravasation of blood and the forming of a hematoma in the fracture gap. As part of blood coagulation, connective tissue is formed that establishes a first elastic connection in the fracture. The ingrowth of chondrocytes results in callus formation of fibrocartilage [5]. Activated osteoblasts gradually ossify the callus until the regeneration is completed.

This process is fraught with problems if the bone defects exceed a certain size (“critical size defect”), age-related healing disorders, and diabetes [6] or during radiotherapy [7].

The common treatment in such cases is the autologous bone transplantation. Typically, a place for bone removal is necessary; therefore, bony tissue is removed at a healthy site for transplantation into the fracture. The amount of bone that can be obtained and the procedure poses additional risks of pain or possible complications.

Tissue Engineering is an important research field in regenerative medicine and with its methods and therapies to approach limitations and develop new therapies in bone defect treatment.

The definition of a critical size bone defect is the smallest possible defect in bone that will not heal during the lifetime of an organism. For many models, the term critical size bone defect is accepted although it may not be clear whether or not the defect is of the smallest in size. Additionally, the end of an experiment is often determined by the endpoint rather than by the natural lifespan of an organism [8].

The choice of a relevant animal model is vital in the development of bone implants. There is a wide range of animals used for investigating different aspects of bone healing and the integration of tissue engineered implants. Desired properties of a model are reproducibility and the chance to conduct different analysis, while morbidity as well as mortality of the animal should be as low as possible until the end of the experiment.

A common view on bone tissue engineering portrays four vital aspects for bone regeneration. A scaffold that is similar in composition and structure to the natural extracellular environment of the bone. Cells are able to produce new bone matrix and additionally, growth factors that facilitate cell differentiation or attract desirable cell types including blood vessels and finally, a vasculature in order to support cell survival for tissues exceeding a certain size [9].

The advances and current developments of vascularization which is necessary to support the survival of cell-seeded implants that exceed a certain size will be reviewed and discussed in the context of bone regeneration.

## Vascularization

The current state of tissue engineering developed various concepts regarding vascularization that differ significantly in their approach. One strategy is the artificial construction of vessels and vessel systems based for example on 3D-printing or the emerging field of cell sheet engineering.

Another strategy is the utilization of controlled but natural vessel formation. This includes the development of the arteriovenous loop (AV loop) grafts and the use of naturally derived decellularized tissue including the vessel network.

## Cell Sheet Engineering

The extracellular matrix plays a major role in the formation of vascular structures. This is an aspect which is often lacking in approaches based on synthetic scaffolds while the scaffold-free cell sheet engineering, similar to the techniques employing decellularized scaffolds, takes this into account. It aims at the constitution and preservation of a rich extracellular matrix. There are different strategies in this approach that make use of different cell types depending on the desired tissue. There are works on treatment of skin damage [10], cornea [11], myocardium [12], bladder [13], or periodontal ligament [14].

An early attempt of cell sheet based constructs is the treatment of burns with epithelial cell sheets [15]. Confluent high density cell layers are used for this technique; they are detached from the cell culture surface resulting in a cell sheet with preserved extracellular matrix which contributes to rapid integration into host organisms after implantation. The gentle detachment of the confluent cell layers can be achieved by temperature sensitive cell culture substrates. This is possible by coating of cell culture surfaces with the thermo-responsive polymer, poly(N-isopropylacrylamide) (PIPAAm). The coated surfaces are hydrophobic at a temperature of 37 °C and allow cells to attach. At a temperature of 32 °C, the surface becomes hydrophilic and fast hydration of the PIPAAm leads to detachment of the cells without interfering with cell-cell contacts or the secreted extracellular matrix. In consequence, differentiated functions of cells are preserved [16].

Regarding bone regeneration, Pirraco et al. combined cell sheets from rat bone marrow stromal cells treated with osteogenic medium with HUVECs seeded in between the sheets in a coculture. The resulting construct was implanted into immunodeficient nude mice and their osteogenic potential analyzed. The histological analyses showed organized bone and increased blood vessel formation 6 weeks after implantation [17].

While there are different mechanisms for bone repair and the periosteum is not contributing to all of them, it is a major aspect regarding bone regeneration and healing. An advanced approach combined the cell sheet technology in order to mimic vessel-rich periosteal tissue with solid ß-TCP cylinders. The periosteum consists of a fibrous outer layer that causes the mechanical stability; it is traversed by blood vessels supplying bone and skeletal muscle. In contrast, the inner cambium layer of the periosteum contains mainly mesenchymal progenitor cells, differentiated osteogenic cells as well as osteoblasts and fibroblasts [18, 19]. With its prominent position in the bone environment, the periosteum takes part in the membranous ossification and the endochondral ossification. The beneficial attributes taken into account, the group of Kang et al. designed an approach that combines the osteoconductive properties of porous ß-TCP cylinders and the supportive functions of cell sheets that form a vasculature and the progenitor cell niche that is usually present in the periosteum. The cylindrical ß-TCP scaffold was covered with a cell sheet composed of two separate layers. The inner layer was a mineralized human mesenchymal progenitor cell sheet, mimicking the cambium. The outer layer was an undifferentiated or osteogenic differentiated hMSC cell sheet seeded with HUVECs on top for 14 days. The coculture formed a vessel-like network during the subsequent 7 days. Immunohistochemical characterization of the vascularized scaffold indicated a rich network of capillaries with cell-lined lumens. The combination of cell sheets wrapped around the ß-TCP cylinders was studied for up to 14 days. Over the course of this time, HUVECs migrate from the sheet onto the scaffold and into the peripheral and central areas. In vivo testing of the combined scaffold in nude mice demonstrates significantly higher vessel densities in implants than in the control group of plain ß-TCP cylinders for week 2 and 4, while the difference vanishes over time until there is no significant difference in vessel densities at week 8. This indicates an accelerated integration of the scaffold into the host tissue and underlines the advantageous effect of the periosteum-like cell sheets. The quantification of the mineral volume revealed a small decrease of volume in the plain scaffold, and the scaffold wrapped in HUVECs on undifferentiated MSC on the cambium layer and a fibrous layer on the outside of the cell sheet. However, the periosteum-like construct with HUVECs on a layer of differentiated MSC and a fibrous layer on the inside, exhibits an increase of approx. 5.5 % in mineralization at week 8. These results confirm the pronounced importance of endothelial cells in tissue engineered grafts for accelerated integration and improved regeneration potential [20•].

## 3D-Printing

With the advent of cell and whole organ culture [21, 22], there was soon the realization of a need to mimic the circulatory system with artificial materials that have no adverse effect on the biological function of the tissue in question [23]. Cardiovascular diseases, accounting for approximately 40 % of all deaths until 1950 [24], demanded special attention in research. The first artificial circulation system as a therapeutic measure after complex surgery was applied by DeBakey in 1966 [25]. In this case, a partially extracorporeal circulatory system with polyester as blood contacting material was used continuously for 10 days. Additional experiments with more advanced structured polyester, polyurethane, and polytetrafluoroethylene surfaces were successfully undertaken in the following decades [26–28].

The research in biomimetic materials led to optimization of the hemocompatibility of materials [29–31]. This was achieved through coating the surface with proteins [32], directly ECM-derived [33], like fibronectin, laminin, collagens or other proteins and molecules with specific binding sites for ECM-associated proteins, e.g., chondroitin sulfate [33] or with pharmacological antithrombogenic potential, e.g., heparin [34], aspirin, and theophylline [35]. Simultaneously, the manufacturing capabilities grew in complexity, with advances in miniaturization of structures and free forming both possible through different additive manufacturing techniques. For vascular grafts electrospinning [36, 37] of (bio-)polymers and multiphotonpolymerization [38, 39•], proved to be useful.

From positive-template grafts to negative-template structures, shortly after its invention in 1989 [40], 3D powder printing was used to generate scaffold structures with controlled micro- and macrostructure from metal [41], ceramic [42], and polymer [43] powders with suitable binder fluids, e.g., phosphoric acid [44] and acrylate dispersions [41]. With this extensive control over the complete internal structure of the printed object, the idea to integrate specific, bigger cavities into the already porous construct was quickly realized. These cavities could be used to control and guide the desired angio- and vasculogenesis to connect the construct to the host circulatory system. With the freedom in shape comes the additional possibility to modify the construct in controlled areas with a wide array of chemical, biological, and pharmaceutical agents, ranging from biologically active metal ions, to the aforementioned ECM-proteins [45, 46] and growth factors like BMP-2 [47] to drugs with antibiotic or anti-inflammatory effects. Some scaffold materials, mostly polymers or peptides, exhibit intrinsic angiogenic induction [48, 49].

Made-to-measure bone implants via solid free form processes are the current state of the art in craniomaxillofacial surgery [50, 51] and generally for small size bone defects. Apart from the aforementioned 3D powder printing, some newer methods, based on typical bone matrix constituents dispersed in hydrogels, have been devised in recent years. Ranging from simple combinations of hydrogels from different polymers [52], with different constituents to bioprinted multicomponent [53] hydrogels with several property gradients, to cryostructured or freeze-/critical-point-dried, xerogels [54], a lot of different and promising techniques and material combinations have been designed and tested.

The major advantage of 3D-printing—having complete control over the geometry and composition of the printed scaffold—was made use of in recent in vivo experiments that created scaffolds, based on calcium phosphate and collagen [55] or polycaprolactone (PCL), collagen and alginate [56•]. In the first case, a commercially available 3D printer was modified to print a calcium phosphate scaffold with confirmed osteoconductive properties. Focus of this study was the optimization of material parameters and the practicability of the use of inkjet based bone substitute creation in a preclinical setting [55]. In the second approach, PCL was building the framework of the scaffold while the collagen type I hydrogel was BMP-2-loaded and co-printed in the peripheral areas of the scaffold. The alginate hydrogel was mixed with VEGF and added to the central areas of the PCL scaffold in order to facilitate vascularization in the potentially hypoxic zone of the implant. Before printing, both hydrogels were supplemented with human dental pulp stem cells that have osteogenic and vascularization potential [57]. The layer-by-layer process of the 3D-printing process allowed for the controlled incorporation of growth factors and cells at the specific sites within the construct. In vivo testing was performed via implantation into the back of 30 mice and results suggested a significant impact of the introduced growth factors as well as cells. Fast vascularization in the center of the implant was observed as well as an associated bone formation along the vascularized structures [56•].

## AV Loop

The arteriovenous loop was first described by Erol and Spira in 1979 and this technique was further developed and is still under investigation today. The AV loop is an in vivo model of axial vascularization and was successfully applied in small as well as large animals [58–60].

The AV loop can be used to vascularize various tissues and materials by placing them in close vicinity to a prominent vessel of adequate size in a so called isolation chamber in uninjured areas of the host’s body. The chamber creates some degree of autonomy of the created biovascularized neotissue graft [61].

Historically, the use of the AV loop in the context of bone regeneration focused on osteogenesis while the aspect of vascularization within the bone was investigated later on. In a large animal study, the successful employment of the methods of AV loop graft generation in sheep is presented [58]. It is demonstrated that the natural diffusion range could be increased in bone substitute materials such as TCP or hydroxyapatite in granular form as well as in solid form of relevant dimensions [62]. An increase in perfusion density was observed for 12 weeks by MRI before the endpoint analyses. Vascularization increased by sprouting from the main vessel of the AV loop. The vessel count decreased from week 6 to week 12, while the average vessel size increased. Limitations lie in the formation of new bone material which could not be confirmed by histological means in this configuration. Additionally, the feasibility of the procedure was demonstrated in standard surgical context. The method was further refined by investigating the effect of the combination of intrinsic and extrinsic vascularization within the vascularization chamber [59•]. The difference in both setups is the isolation chamber which was accessible by the surrounding tissue through perforation of the titanium chamber, so that vascularization was possible along the AV loop utilized (intrinsic vascularization) as well as through the surrounding area. The exclusively intrinsic vascularization was achieved by a Teflon chamber that allowed no external access except for the arteriovenous vessel that was intruded into the chamber. The joint mechanisms of intrinsic and extrinsic vascularization proved to be superior to their single capacities [59•]. An early clinical attempt of vascularizing a bone graft in human host tissue was the implantation of a mandibular bone graft for the restoration of the lower jaw. For in vivo vascularization, the graft consisting of bovine derived bone mineral was mixed with recombinant human BMP7 and bone marrow aspirate. This method avoided creating a second site bone damage but caused donor-site morbidity in the latissimus dorsi muscle that served as a basis for graft vascularization [63]. Further clinical applications of the AV loop technique were described for a tibial defect and a large bone defect in the wrist due to carcinoma removal and it could contribute to the healing of those defects that previously did not heal without further intervention. The technique was modified in a way that the AV loop was created directly in the defect site, rendering a donor-site redundant and thus, eliminating a major issue at least for specific cases since not all areas are eligible for this procedure [64•].

## Naturally Derived Decellularized Scaffolds

Native bone as it is used in autologous bone transplants is the golden standard for the treatment of large defects. The critical disadvantages of this approach can be countered by the use of allogenic or xenogenic bone that has been decellularized. Among others, an important factor for the osteoconductive effect of materials used for bone regeneration is the micro- and macrostructure, e.g., the pore shape and size, the geometry as well as micropores and roughness of the surface [65]. All of these aspects can be utilized by decellularizing bone harvested from allogenic or xenogenic sources and thus nullifying the disadvantage of limited availability. During the essential removal of all cellular components, the bone extracellular matrix also loses many of its biologically active signals [66] while it still remains a potent biomaterial. A reintroduction of cues facilitating bone regeneration is possible via seeding decellularized bone with autologous cells or the addition of growth factors and thus, restoring a great deal of regenerative potential.

Decellularized vascular structures are a promising component of the tissue engineering approach at supporting cells and tissues of a regenerative implant that exceeds the dimensions that can be supported by diffusion alone with adequate amounts of nutrition and oxygen. Decellularized tissues are composed of a biocompatible extracellular matrix that naturally possesses mechanical properties suitable for the application of the intended purpose. The extracellular matrix itself can have a critical role in the regeneration of different tissues [67] and additionally, it can be reseeded with human primary cells of endothelial linage to restore functionality of a vessel system that is capable of providing nutrition for other cell types in coculture. An example is the reintroduction of microvascular endothelial cells into the vascular structures of decellularized porcine jejunum of the small intestine [68]. The repopulation with endothelial cells reduces the probability of thrombus formation and the calcification of vessels [69]. Reseeded vascular structures can be dynamically perfused in specifically designed bioreactor systems in order to preserve the characteristics of differentiated endothelial cells or to initiate the differentiation of endothelial progenitor cells by applying shear stress [70]. It was shown that such a vascular graft can support survival of three-dimensional tissue of considerable size. Such a prevascularized scaffold was filled with hepatocytes in the former lumen and a complex, structured liver-like tissue was formed. It was supported by capillaries sprouted from existing small vessels of the wall of the jejunum into the lumen [71]. This principle was transferred to the creation of a prevascularized bone implant (data not published). The vascularization scaffold was combined with synthetic tricalcium phosphate seeded with bone marrow-derived mesenchymal progenitor cells. The material provides osteoconductive properties and serves as a scaffold for the mesenchymal progenitor cells which are known to contribute to bone regeneration in many ways [72]. After a dynamic bioreactor-driven coculture of endothelial progenitor cells in the vascularization scaffold and the cell-seeded bone substitute, the whole construct was used in two different large animal studies in sheep for bone regeneration of a large tibia defect as well as a continuity defect in the mandible. The studies are described in a later chapter (“Animal models for bone regeneration”).

## Animal Models for Bone Regeneration

There is a range of animal models available for bone regeneration and bone substitute material development [8, 73–75]. Optimally, a suitable model should be reproducible, allow several types of analysis, is versatile regarding investigated materials or strategies. Influence of age and sex on bone regeneration through calciotrophic factors have to be considered [76, 77]. The model should allow the generation of sufficient data, morbidity should be low and the costs reasonable [78].

Depending on the aspect of investigation, different animal models come into consideration. The most common species are rodents (mice and rats) that make up more than three-fourths, while pigs, rabbits, dogs, small ruminants, and non-human primates represent the remaining model organisms [79].

The merits of using rodents is easy handling as well as having high numbers to generate statistically significant data. Especially mice allow for specific or conditional knockout studies. They are suitable for investigating osteoinductive materials from allogenic or xenogeneic sources since athymic nude mice can be readily utilized if the immunogenicity of a tested material is unclear [79]. Important considerations for the comparison to human bone regeneration are differences in general bone healing potential and differences in the micro- and macrostructure like the ratio of cortical and cancellous bone that determines the biomechanical properties of the specific bone investigated [80, 81]. Mice and rodents have open growth plates in the epiphysis throughout their experimental life time, so they retain longitudinal growth, a capability that is not available for adult humans [82].

In contrast to rodents, rabbits have a Haversian system and allow studying bone regeneration with closed growth plates which reflects the human condition better. Bone densities of rabbits and humans are similar; however, shape and size of rabbit bones differ from the human anatomy and limit studies focusing and mechanical properties [83]. Among others, this species is commonly applied to studies dealing with cranial [84] and mandible defects [85] and age-related critical size defects [86]. To this end, autologous cell-seeded or cell-free biomaterials are used as well as growth factors or the direct application of stem cells [87] or negative pressure [88].

Another frequently used model is the canine model, especially for periodontal and musculoskeletal research [89]. Additionally, it finds application as cranial model [90] and for investigation of the regenerative potential of adipose-derived or bone marrow stem cells and biomaterials as well as the effect of various growth factors and corresponding dose-response studies [91, 92]. The dog’s bone is similar to the human’s in respect of structure, composition, and remodeling rate [93]. The size of large dogs allows for the evaluation of implants of a size suitable for humans. The use of dogs as model is often critically evaluated due to low social acceptance [94].

Similar to dogs, pigs have a bone regeneration process comparable to the one in humans. The bone morphology, bone structure and composition together with remodeling closely resembles the human condition [95•, 96], although there are differences regarding the density of the trabecular network and an increased strength [97, 98]. The model is used for investigations regarding bone fracture healing and femoral head necrosis regulatory toxicity studies [93, 99]. The pig was a popular model for the testing of bone substitutes in critical size defect due to their healing rate which matches that of a human [95•]. Potential disadvantages and shortcomings of commercial pigs like high growth rate and body weight, challenging manageability have been overcome by the development of mini and micro pigs [100].

Sheep and goats are associated with more convenient handling, animal cost, and social acceptance as animal model [101]. Their anatomy is similar to that of humans regarding long bones of suitable dimensions for supporting implants of a size that could be used in humans [102, 103]. Analogous to other large animals, the bone macrostructure of sheep and goats are comparable to that of humans. The fact that sheep bone mainly consist of primary bone in contrast to mostly secondary bone in humans, can be accounted for by using sheep of an older age (7–9 years) when the secondary bone remodeling occurs [102]. Despite differences in the bone microstructure, the value of sheep and goats is the modeling of human bone turnover and remodeling [104]. They have been used for segmental defect models [105], vertebral bone defect models including studies testing bone substitute materials [106], the effect of growth factors [107], and tissue engineering strategies [73].

Despite the convenience of small animals as models, certain scenarios necessitate studies to be conducted in large animals, for example in order to study their feasibility or implants whose size will not allow small animals as model, when long diffusion distances are investigated as well as transport limitations that might affect survival of cell-seeded implants. Sophisticated surgical procedures might limit practicability in clinical routine. Larger animals offer more comparable data regarding biomechanical analysis and evaluating the feasibility of the surgeries necessary. In the EU project “VascuBone” (Grant Agreement 242175) the consortium tested a synthetic bone substitute material prevascularized by the combination of a naturally derived collagen scaffold as described above (chapter “Naturally derived decellularized scaffolds”) in an ovine segmental bone defect according to Reichert et al. [73] and a newly developed ovine mandibular continuity defect. In case of the tibia defect, the construct was implanted by Hutmacher group [108, 109] and used to bridge a 3-cm defect resulting from bone excision. The vascularization scaffold was pulled over the proximal and distal ends and sutured to the remaining periosteum and the defect was stabilized with a DCP plate.

The mandible defect is a clinically relevant scenario for ablative tumor resection or osteonecrosis caused by radiation or drugs as bisphosphonates in the maxillofacial context. Rasse and Stigler (Clinical Department of Craniomaxillofacial and Oral Surgery, Medical University of Innsbruck) excised in the jaw angle below the musculus masseter and stabilized the defect with two titan plates and an additional titan cage. The prevascularized implant featured an artery and vein that were anastomosed with the host’s blood circulation via the facial transverse artery. Preliminary results (not published) showed good integration of the implant and bridging of the defect after 6 months. This indicates the importance of the implant’s vascular support and anastomosis as the control group featuring the same scaffold and material without cells and no anastomosis showed reduced bone regeneration. The empty defect displayed no regeneration at all. The positive performance of the presented model shows the possibility of the translation into a large animal model which is an important prerequisite for clinical trials.

## Conclusion

The broad range of available animal models is confronted with an even broader range of scientific questions. No animal model represents the human condition in its entirety; hence it is important for the experimental design to evaluate the qualification of the possible models for the question at hand in order to find the most suitable model. At present, many studies deal with comparable problems that are investigated in models that differ significantly so that the emerging results do not allow valid conclusions which approach at the same problem is more beneficial. Thus, a general consent on the exact use of test animals has to be striven for. There is already the general tendency to use certain models for specific scenarios: for example, mouse models for investigations that necessitate genetic manipulation or larger animals for the biomechanical studies. Nevertheless, many details in the general routine of animal experimentation are not standardized and should be harmonized within the scientific community in order to make the most out of the valuable data that can be acquired from animal experimentation. Additionally, the predictability of models has to be increased by developing new or refined model systems.
